# A new automated method for analysis of rCBF-SPECT images based on the active-shape algorithm: normal values

**DOI:** 10.1111/j.1475-097X.2011.01063.x

**Published:** 2012-03

**Authors:** Douglas Hägerström, David Jakobsson, Erik Stomrud, Ann-Margret Andersson, Erik Ryding, Elisabet Londos, Lennart Minthon, Ingmar Rosén, Lars Edenbrandt

**Affiliations:** 1 Clinical Neurophysiology Unit, Department of Clinical Sciences Lund, Lund UniversityLund; 2 Department of Clinical Neurophysiology, Skåne University HospitalLund; 3 EXINI diagnostics ABLund; 4 Clinical Memory Research Unit, Department of Clinical Sciences Malmö, Lund UniversityMalmö; 5 Clinical Physiology Unit, Department of Clinical Sciences Malmö, Lund UniversityMalmö; 6 Department of Molecular and Clinical Medicine, Sahlgrenska University Hospital, University of GothenburgGothenburg, Sweden

**Keywords:** active shape, image processing, normal values, quantification, radionuclide imaging, regional cerebral blood flow, single-photon emission computed tomography

## Abstract

Most nuclear medicine clinicians use only visual assessment when interpreting regional cerebral blood flow (rCBF) from single-photon emission computed tomography (SPECT) images in clinical practice. The aims of this study were to develop a new, easy to use, automated method for quantification of rCBF-SPECT and to create normal values by using the method on a normal population. We developed a 3-dimensional method based on a brain-shaped model and the active-shape algorithm. The method defines the surface shape of the brain and then projects the maximum counts 0–1·5 cm deep for designated surface points. These surface projection values are divided into cortical regions representing the different lobes and presented relative to the whole cortex, cerebellum or cerebellar maximum. ^99m^Tc-hexa methyl propylene amine oxime (HMPAO) SPECT was performed on 30 healthy volunteers with a mean age of 74 years (range 64–98). The ability of the active-shape algorithm to define the shape of the brain was satisfactory when visually scrutinized. The results of the quantification show rCBF values in the frontal, temporal and parietal lobes of 87–88% using cerebellum as the reference. There were no significant differences in normal rCBF values between male and female subjects and only a weak relation between rCBF and age. In conclusion, our new automated method was able to quantify rCBF-SPECT images and create normal values in ranges as expected. Further studies are needed to assess the clinical value of this method and the normal values.

## Introduction

Regional cerebral blood flow (rCBF) measurement has been a part of the extended clinical investigation of dementias for decades and since the beginning of the 1990s mainly with ^99m^Tc-hexa methyl propylene amine oxime (HMPAO) or ^99m^Tc-ethyl cysteinate dimer. Quantification of rCBF images has its natural place in clinical neuro-psychiatric research and there is an increasing number of commercially available quantification methods on the market, for example, SPM, Hermes BRASS and NeuroGam. However, the use of those in clinical practice is not widely spread. Manual or semi-automated methods for quantification can be laborious and time-consuming to use and it can also result in lack of reproducibility if many operators are involved in a clinical setting. Such methods are therefore not suitable for clinical routine use. Instead most nuclear medicine physicians use only visual assessment when interpreting rCBF-SPECT images despite that considerable inter-observer variation has been reported (Honda *et al.*, 2002). Reports, based on visual assessment only, have a tendency to be more vague and unstructured compared to those based also on quantitative data and this is probably one of the reasons for the limited use of rCBF-SPECT by neurological or psycho geriatric clinicians in clinical routine investigations of dementia. Quantitative analysis as a complement to visual assessment will probably make the assessment of functional abnormalities more reliable and the interpretations more objective.

The need for accurate diagnostic procedures will increase as the clinical focus is shifting from diagnosis of Alzheimer's disease (AD) to the prediction of conversion from mild cognitive impairment to AD. Patients with less severe disease will be examined and the interpretation will be more difficult. Quantitative analysis of CBF-SPECT could play an important role in this diagnostic challenge.

The aims of this study were to develop a new, easy to use, automated method for the quantification of rCBF-SPECT, and to create normal values by applying the method on a normal population.

## Methods

### Subjects

The normal group consisted of 30 healthy volunteers, 20 women and 10 men, with a mean age of 74·4 (SD 7·8; range 64–98). The subjects were recruited as cognitively healthy controls in a dementia study at the Neuropsychiatric Clinic, Malmö, Sweden (Stomrud *et al.*, 2007, 2010). Sixty-two individuals were included in the study at baseline in 2002. All subjects underwent a comprehensive work-up, including medical and psychiatric examination, blood and cerebrospinal fluid collection, electrocardiogram, computed tomography, orthostatic test and cognitive tests. Cognitive follow-ups were performed after three and 5 years. At the follow-up after 5 years, rCBF-SPECT examinations were performed on the 40 subjects that had not been excluded during the follow-up period. Cognitive testing was performed with the Mini-Mental Status Examination and the Alzheimer Disease Assessment Scale Cognitive Subscale.

Inclusion criteria were intact cognitive function and unaffected activities of daily living functions during the follow-up period. Exclusion criteria were physical or mental disease, affecting cognitive status, and fulfilment of the criteria for any dementia disorder or Mild Cognitive Impairment.

A visual evaluation of all rCBF-SPECT examinations was performed by two experienced physicians to exclude subjects with obvious focal abnormalities, such as small vascular lesions. This exclusion process was not based on any quantitative analysis. Ten subjects were excluded in this visual evaluation.

A separate group of rCBF-SPECT examinations from 29 patients, 17 women and 12 men, with a mean age of 49·4 (SD 12·6; range 32–75) was used in the development of the brain-shaped model (see Automated method below). The selection of these rCBF-SPECT examinations was based on the following criteria:

1. Images with high quality.
2. Easy to see the transition between the brain surface and the background.
3. Minimal uptake outside the brain interfering with the brain surface.
4. Includes small, medium-sized and large brains.
5. Includes both female and male patients.

The study was approved by the regional ethics committee at Lund University.

### Image acquisition

Intravenous injection of 900 MBq ^99m^Tc-HMPAO was performed in silence, apart from ambient noise, in a well-lit room, with the patients resting supine, quiet and with open eyes. Data acquisition began about 30 min later using a dual-head gamma camera (Siemens Symbia T2; Siemens Medical Solutions, Knoxville, TN, USA), equipped with low-energy high-resolution collimators, rotating full 360° in an optimally close, non-circular orbit, with 128 projections per detector head. The recordings were reconstructed to a 128 × 128 × 128 matrix using 3D-OSEM algorithm (Flash 3D; Siemens Medical Solutions) with DEW scatter correction and attenuation correction based on a sequentially performed low-dose CT scanning. The spatial resolution was approximately 10 mm (Full Width at Half Maximum).

The 29 patients in the separate group used for the development of the brain-shaped model were examined using a 3-headed gamma camera (MultiSPECT 3; Siemens Medical Solutions). The image reconstruction was performed with filtered back-projection, attenuation was modelled to be uniform inside the skull volume, and no scatter subtraction was performed. Image acquisition was otherwise the same as for the normal subjects.

### Automated method

The new method for the quantification of rCBF-SPECT images is based on the active-shape algorithm described by Cootes & Taylor (2001). The brain is segmented in 3-dimensions in an iterative process using a brain-shaped model.

The model, which contains statistical information of the variability of brain shape in the 29 selected rCBF-SPECT patient examinations, was obtained in the following way: Landmarks were manually marked in all sagittal slice images of the 29 rCBF-SPECT examinations to form closed curves marking the brain surface. The sagittal view was considered to be the best choice, as the brain surface can be enclosed with one single curve in all slices, which is not the case for the transaxial and coronal views. The landmarks were arranged to a total of 546 coordinates per patient examination with fixed positions on the brain surface by the use of interpolation. The 29 manually defined brain shapes were then aligned to a common coordinate frame. The mean shape and the covariation matrix of the landmarks were calculated. The shape variation was modelled by calculating the eigenvectors and the corresponding eigenvalues of the covariation matrix. This procedure is commonly known as Principal Component Analysis (PCA). The five eigenvectors with largest eigenvalues were chosen. These five eigenvectors, called modes, can be used to approximate any of the brain shapes in the training group or to generate similar new brain shapes by using different parameters for each mode.

Each landmark was assigned to one of the following brain lobes:

1. Cerebellum (total, dx, sin).
2. Frontal lobe (median part, dx, sin).
3. Temporal lobe (dx, sin, median part dx, median part sin).
4. Parietal lobe (dx, sin).
5. Occipital lobe.

This was achieved with the help of brain SPECT phantom images based on MRI data. The phantom images were created using the SIMIND Monte Carlo program (Ljungberg & Strand, 1989). This program makes it possible to create images with uptake in one brain lobe only and with zero uptake in the rest of the brain that greatly facilitated the division of the landmarks into lobes. The result was inspected and adjusted visually. Finally, the division of the landmarks of the left hemisphere was mirrored to the right hemisphere, resulting in a symmetrical brain atlas.

With this brain model, new rCBF-SPECT images can be analysed in five steps described below. The system require that the images are acquired using a matrix size of 128 × 128 or 256 × 256.

#### Step 1 – Rotation

The transaxial slice images from new rCBF-SPECT images are used as input. An algorithm for automatic rotation of the image volume in the sagittal view is applied. PCA was used to decide the angle for which the brain tissue has the largest extension. The angle was used in combination with an offset angle to rotate the image volume so that the lowest part of the frontal lobe was approximately horizontal with the intersection of the cerebellum and occipital lobe. The rotation of the image volume could be corrected manually if required.

#### Step 2 – Delineation of brain surface

The brain model is fitted to new rCBF-SPECT images by using the active-shape algorithm in the following way:

A search is performed for the first estimate of the brain surface by scaling, translating and rotating the brain model to fit the brain surface of the new image volume. This estimate is used as starting point for the iterative process, in which the brain-shaped model is adjusted to optimize the fit with the image data. The algorithm searches for new points along the normal vectors of each point. A point was moved if a new point was found that better matches the characteristics of the brain surface. These criteria were based on the intensity interval and the intensity profile that are typical for voxels on the boundary of the brain tissue and background. The shape model was fitted to the new points, and the procedure was iterated until convergence. The result of the delineation process was visually inspected and approved for each rCBF-SPECT examination.

#### Step 3 – Normalization

A count normalization was performed so that the voxel intensity values within the image volume were normalized to each of three reference values:

1. The mean value of the cortical part of cerebellum.
2. The maximum intensity value of cerebellum (the mean of the maximum intensity value and the intensity values of the surrounding 26 voxels).
3. The mean value of the whole cortex.

The reference value was put to 100% and all voxel intensity values were related to that.

#### Step 4 – Calculation of the cortical blood flow

In each point at the brain surface, a count profile perpendicular to the surface of the model is extracted. For each profile, the maximal count value from the brain surface and for a distance corresponding to 9% of the length of the brain into the cortical matter is noted. This distance corresponds to approximately 15 mm for a human brain of normal length. The maximum value of each intensity profile was used as an estimate of cortical blood flow for that sample point. A total of 3010 sample points were used in the quantification of the cortical blood flow. These sample points were interpolated from the 546 landmarks used in the delineation of the brain surface. The mean value of the sample points in a region was used as an estimate for the mean rCBF in that region.

The difference between the mean cortical blood flow of the left (CBF_sin_) and right (CBF_dx_) side of a region was calculated as follows:


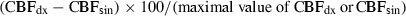


#### Step 5 – Calculation of the cortical index

A cortical index was calculated to reflect the ratio between voxels with high intensities (e.g. normal cortical tissue) and voxels with low intensities (e.g. ventricles) within the brain volume. High intensities were defined as values of at least 45% of the reference value.

### Calculation of normal values

Mean values and standard deviations for the 30 normal subjects were calculated for each sample point on the brain surface, each region and for the cortical index. The calculations were made separately for each of the three reference values.

### Statistical methods

The significance of differences in mean CBF between male and female subjects was tested using a *t*-test for unequal sample sizes and unequal variance. The significance of differences in mean CBF between left and right lobes was tested using a dependent *t*-test for paired samples. The Pearson product-moment correlation coefficient (*r*) was used as a measure of the correlation (linear dependence) between CBF values and age. The significance level was set to <0·05. Results

## Results

The ability of the active-shape algorithm to define the shape of the brain was satisfactory in all 30 subjects when visually scrutinized. Only 440 of the 90 300 (0·49%) sample points (3010 sample points per patient; 30 patients) used in the quantification of rCBF showed values <45% of the reference value, indicating a possible false delineation of the brain. The processing time for a rCBF-SPECT was <20 s on a standard desktop computer.

**Figure 1:**
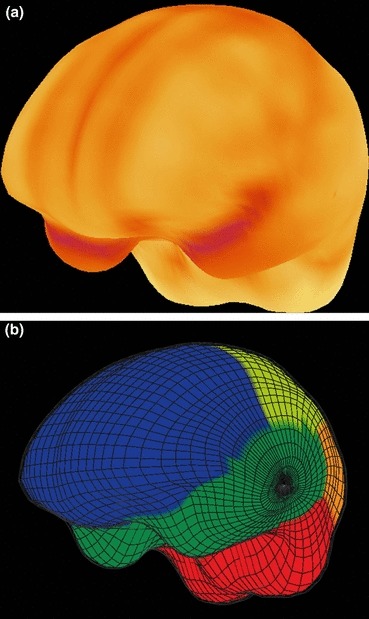
A freely rotatable 3-dimensional brain model showing the mean regional cerebral blood flow (rCBF) in each surface point for the normal subjects (a) and the same model showing the anatomical regions used in the quantification (b).

Normal rCBF values for the brain regions, normalized to each of the three reference regions, are presented in Table 1 and Fig. 1. The cortical index was on average 63·1% (SD ±7·2%). There were no significant differences in normal rCBF values between male and female subjects. The left sides of the cerebellum and parietal lobe showed significantly higher mean rCBF compared to the right side. There were no significant side differences in the temporal and frontal lobes.

**Table 1:** Normal rCBF values (mean and standard deviations) for the three normalization methods (See Methods)

Region	Cerebellum	Normalization point	Cortex
Cerebellum		78·9 + 3·4	111·6 + 3·2
Cerebellum dx		78·1 + 3·3	110·5 + 3·4
Cerebellum sin		79·6 + 3·7	112·6 + 3·5
Frontal lobe dx	86·8 + 3·4	68·5 + 4·3	96·8 + 1·9
Frontal lobe sin	87·0 + 3·4	68·7 + 4·5	97·0 + 2·0
Frontal lobe med	82·2 + 6·2	64·9 + 6·2	91·7 + 6·6
Temporal lobe dx	87·0 + 3·8	68·6 + 4·6	97·0 + 2·5
Temporal lobe sin	88·2 + 3·6	69·6 + 4·7	98·3 + 2·5
Temporal lobe med dx	77·2 + 3·5	60·9 + 4·3	86·1 + 2·5
Temporal lobe med sin	78·6 + 3·5	62·1 + 4·3	87·7 + 2·9
Parietal lobe dx	87·2 + 3·9	68·9 + 4·7	97·3 + 3·1
Parietal lobe sin	88·5 + 4·4	69·9 + 5·2	98·7 + 3·9
Occipital lobe	97·0 + 3·2	76·6 + 5·0	108·2 + 2·5

rCBF, regional cerebral blood flow.

**Figure 2:**
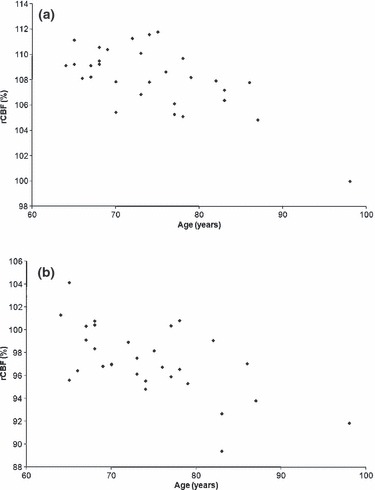
Relation between mean regional cerebral blood flow (rCBF) in the (a) occipital lobe (*r* = −0·66) and (b) right parietal lobe (*r* = 0·061) and age, respectively.

The relation between mean rCBF and age in the occipital and right parietal lobes is presented in Fig. 2. In these regions, a slight decrease in rCBF with age was found (occipital lobe *r* = −0·66, *P*<0·001; right parietal lobe *r* = −0·61, *P*<0·001). In other regions, the absolute *r*-values were <0·4.

## Discussion

We have developed a new automated method for the quantification of rCBF-SPECT. The innovative approach with our method is the use of the active-shape algorithm. With this technique, a brain-shaped model is fitted to the patient's rCBF-SPECT images instead of, for example, fitting the patient image to the Talairach idealized brain. The actual shape of the patient's brain can be preserved in the images instead of being distorted. The active-shape method has previously been applied to myocardial perfusion scintigraphy for the delineation of the left ventricle (Lomsky *et al.*, 2005). The method is also completely automated and not time-consuming for the user with a processing time of <20 s on a standard desktop computer.

We did only find a weak relation between rCBF and age. This finding is in contrast to that of Catafau *et al.* (1996) who found lower rCBF in aged (mean age 71 years) compared to young (mean age 29 years) volunteers. Krausz *et al.* (1998) also found lower rCBF in their older subjects (47–71 years) compared to their younger subjects (26–47 years). The reason that we only found weak age-related changes can be that our study group was older than in these studies. Mozley *et al.* (1997) showed that most of age-related changes occur during young adulthood. They found that the rate of change became negligible after 36 years of age, i.e. at a much lower age than those of the subjects included in this study. Catafau and colleagues did not find any significant rCBF differences between the genders and that is in agreement with the results of our study. Comparisons with other studies regarding rCBF in different regions of the brain are not possible because of the differences in quantification methods and definitions of regions of interests.

The automated method is designed to assess the cortical blood flow as this has showed to be a valuable parameter for the diagnosis of AD. Tang and colleagues showed that 3-dimensional surface projections showing cortical blood flow was significantly better than 2-dimensional slice images in terms of reproducibility and diagnostic performance in respect of AD in patients with cognitive impairment and they concluded that it provides a valid tool for assessment of the severity of cortical perfusion abnormalities in such patients (Tang *et al.*, 2004). In this study, the 3D-SSP method presented by Minoshima *et al.* (1995).

The selection of the reference region is important in quantification and display of rCBF-SPECT images as absolute flow values are not available. The whole brain and the cerebellum are the most common references used, but they do not always represent the best choice. The reference region should ideally have a normal blood flow and in patients with reduced blood flow in these regions the quantitative data may be inaccurate. For a large reference region such as the whole cortex, the problem will not be substantial if the abnormal parts are small, but large abnormalities may be underestimated because they will decrease the reference value. As an alternative reference region to be used in patients, where the other two reference regions are judged to be unreliable, the maximum value of cerebellum was added to the quantification method.

The definition of normality is difficult and raises practical problems, for example, in the choice of method to include and exclude subjects in the development of normal values for a diagnostic examination. In general, two types of normal populations are considered: patients with normal findings in one or more diagnostic examinations and normal volunteers with low likelihood of disease. The use of patients with normal findings can be criticized as the subjects referred to an examination do have some reasons for the referral, indicating that they may not be representative of a healthy population. Such patients can be supposed to represent a ‘more sick’ part of the healthy population, leading to excessively broad normal limits for normality. The use of normal volunteers is also difficult because subjects with subtle symptoms may see this type of study as an opportunity to have a health-check. This type of subjects may also represent a ‘more sick’ part of the healthy population. To avoid the inclusion of individuals with obvious abnormalities in a normal population, it is common to include a process in which one or more experts exclude typically abnormal examinations. However, it is important that these experts do not have a too sensitive interpretive style. If they classify even small or subtle changes as abnormal, this may lead to a supernormal population, leading to too narrow normal limits. We believe that the inclusion and exclusion criteria in this study represent a reasonable balance to have a relevant normal population for establishing normal limits.

The normal values also depend on, for example, the imaging procedure, reconstruction technique, scatter and attenuation correction. In this study, the subjects had open eyes at the time of injection, the images were reconstructed using a 3D-OSEM algorithm, and a low-dose CT scanning was used for scatter and attenuation correction. This always has to be considered before using normal values in clinical routine.
